# Factors Associated with Arkansans' First Use of Telehealth during the COVID-19 Pandemic

**DOI:** 10.1155/2022/5953027

**Published:** 2022-06-28

**Authors:** Jennifer A. Andersen, Holly C. Felix, Dejun Su, James P. Selig, Shawn Ratcliff, Pearl A. McElfish

**Affiliations:** ^1^College of Medicine, University of Arkansas for Medical Sciences Northwest, 1125 N. College Ave., Fayetteville, AR 72703, USA; ^2^Fay W. Boozman College of Public Health, University of Arkansas for Medical Sciences, 4301 W. Markham St., Little Rock, AR 72205, USA; ^3^Center for Reducing Health Disparities, College of Public Health, University of Nebraska Medical Center, 984340 Nebraska Medical Center, Omaha, NE 68198, USA; ^4^Department of Health Promotion, Social & Behavioral Health, University of Nebraska Medical Center, 986075 Nebraska Medical Center, Omaha, NE 68198, USA; ^5^Fay W. Boozman College of Public Health, University of Arkansas for Medical Sciences Northwest, 1125 N. College Ave., Fayetteville, AR 72703, USA; ^6^Department of Sociology, University of Nebraska-Lincoln, 711 Oldfather Hall, Lincoln, NE 68588, USA

## Abstract

**Objective:**

To examine the factors associated with the first use of telehealth during the COVID-19 pandemic using Andersen's Model of Healthcare Utilization. Andersen's Model of Healthcare Utilization allowed the categorization of the independent variables into the following: (1) predisposing factors, including sociodemographic variables and health beliefs; (2) enabling factors, including socioeconomic status and access to care; and (3) need for care, including preexisting or newly diagnosed conditions and reasons to seek out care or to utilize a new mode of care.

**Methods:**

Potential respondents (*n* = 4,077) were identified for recruitment from a volunteer registry in Arkansas. Recruitment emails provided a study description, the opportunity to verify meeting the study's inclusion criteria and to consent for participation, and a link to follow to complete the survey online. The online survey responses were collected between July and August of 2020 (*n* = 1,137).

**Results:**

Telehealth utilization included two categories: (1) *utilizers* reported the first use of telehealth services during the pandemic, and (2) *nonutilizers* reported they had never used telehealth. Lower odds of reporting telehealth utilization during the pandemic were associated with race (Black; OR = 0.57, CI [0.33, 0.96]) and education (high School or less; OR = 0.45, CI [0.25, 0.83]). Higher odds of reporting telehealth utilization included having more than one provider (OR = 2.33, CI [1.30, 4.18]), more physical (OR = 1.12, CI [1.00, 1.25]) and mental (OR 1.53, CI [1.24, 1.88]) health conditions, and changes in healthcare delivery during the pandemic (OR = 3.49, CI [2.78, 4.38]).

**Conclusions:**

The results illustrate that disparities exist in Arkansans' utilization of telehealth services during the pandemic. Future research should explore the disparities in telehealth utilization and how telehealth may be used to address disparities in care for Black Arkansans and those with low socioeconomic status.

## 1. Introduction

In early 2020, the first cases of COVID-19, the disease caused by a novel coronavirus (SARS-CoV-2), were diagnosed in the United States [1, 2]. To reduce the spread of SARS-CoV-2 and limit the effect of a viral pandemic in the United States, many mitigation efforts were undertaken, including limiting face-to-face healthcare services and increasing the use of telehealth. Even with the return to in-person appointments, healthcare providers have continued more robust telehealth services to meet the need of their patients [3–6]. In the context of a global pandemic, Andersen's Model of Healthcare Utilization (hereafter referred to as Andersen's Model) provides a useful framework for exploring factors that influence telehealth utilization during the COVID-19 pandemic, including factors that are unique to the pandemic itself [7, 8]. These factors include the perceived susceptibility to COVID-19 and pandemic-related fears that may play a role in the decision to use telehealth services. Originally conceptualized for general health services, Andersen's Model consists of three components which may influence telehealth utilization: (1) predisposing factors, including sociodemographic characteristics and health beliefs; (2) enabling factors, including social support, wealth, and access to care; and (3) need for care, including preexisting and newly diagnosed conditions, or other reasons to seek out care [7, 8]. The aim of this study was to identify factors associated with first time telehealth utilization in Arkansas during the COVID-19 pandemic using Andersen's Model.

### 1.1. Predisposing Factors

Telehealth services have not been accessible for all populations prior to COVID-19. For many, telehealth services were not covered by health insurance prior to COVID-19, and access was limited by a lack of authorized reimbursement [9]. Moreover, minority populations, those in lower socioeconomic statuses, older adults, and urban populations are less likely to access telehealth services than their counterparts [10–18]. Prior research has demonstrated that the limited uptake of telehealth in these populations is associated with issues of access (e.g., the availability of broadband internet and internet-capable devices in the home) [10–16]. Further associations have been found between limited telehealth utilization and the lack of individual technical literacy and technical support from family and friends, a lack of trust in the effectiveness of telehealth (e.g., provider not being in the same room to examine patient), and the security of telehealth (e.g., concerns of confidentiality of private information) [10–16].

In addition to the sociodemographic factors, there are specific COVID-19 pandemic-related factors to consider as well. These factors include the need to understand where pandemic-related fears and the perceived susceptibility to COVID-19 may play a role in the decision to use telehealth services, potentially by altering an individual's health beliefs. Therefore, we hypothesize for predisposing factors:

H1a: people who are racial/ethnic minorities, older, female, married, or with lower levels of education will be less likely to use telehealth

H1b: people who have higher perceived susceptibility to COVID-19, higher levels of fear of contracting COVID-19, or more confidence in their medical knowledge will be more likely to use telehealth

### 1.2. Enabling Factors

The COVID-19 pandemic has highlighted existing socioeconomic inequities and disparate access to testing and healthcare for COVID-19 [19–23]. Businesses closing and/or reducing employee work hours, as well as the need for individuals exposed or infected to quarantine/isolate, has led to financial instability and the loss of health insurance [24–26]. Stimulus payments from the federal government provided a source of income, potentially allowing for healthcare access. Prior research has shown that people with a regular primary care provider (PCP) are more likely to use health services than those without a regular PCP [27–29]. Therefore, we hypothesize for enabling factors:

H2a: people who are employed for wages, have insurance coverage, or have received a COVID-19-related stimulus payment will be more likely to use telehealth

H2b: people who have at least one regular healthcare provider will be more likely to use telehealth

### 1.3. Need for Care

Those with a higher risk profile (e.g., preexisting conditions) may face additional barriers to accessing face-to-face care due to the risk of exposure to COVID-19 in medical clinic waiting rooms or the cancellation of surgeries or other vital medical procedures. These COVID-19-related factors may increase the perception of the urgent need for care with limited ways to receive it. Therefore, we hypothesize for need for care:

H3: people with more preexisting conditions, more barriers to face-to-face healthcare, or worsening health during the pandemic will be more likely to use telehealth

To improve telehealth utilization during the current public health crisis and prepare for future crises, it is important to understand the factors associated with an individual's decision to utilize telehealth services [6, 30–32]. Our study sought to identify factors associated with first time telehealth utilization in Arkansas during the COVID-19 pandemic.

## 2. Methods

The study utilized a cross-sectional survey design. The study protocol was approved by the University of Arkansas for Medical Sciences (UAMS) Institutional Review Board (IRB#261226).

### 2.1. Participants

The study's inclusion criteria consisted of being an adult (≥18 years of age) and living, working, or receiving healthcare in Arkansas. Potential respondents with valid email addresses (*n* = 4,077) were identified in the ARresearch registry (*n* = 4,431) for recruitment via email. ARresearch.org, established by the Translational Research Institute at the University of Arkansas for Medical Sciences (UAMS), is a volunteer registry through which individuals sign up to be contacted about research opportunities, including those needing healthy volunteers.

### 2.2. Materials

The variables used for this study were part of a larger survey on the effects of the COVID-19 pandemic on individuals living in the state of Arkansas. The survey was developed by the study team using validated questions drawn from existing instruments and survey questing databases including the Behavior Risk Factor Surveillance Survey and PhenX Toolkit [33, 34], as well as new questions specific to the COVID-19 pandemic. Respondents' names, dates of birth, and email addresses were also collected to identify duplicate survey responses; however, data were deidentified prior to the analysis.

### 2.3. Procedures

Recruitment emails provided a study description, the opportunity to verify meeting the study's inclusion criteria and to consent for participation, and a link to follow to complete the survey. Respondents indicated consent by agreeing to participate in the survey. Data collection ran from July to August 2020. Those completing the survey received a $20 gift card as remuneration.

There were 1,288 survey responses received (response rate ~31%). Eleven surveys were duplicates, in which case, the first survey was retained while the second was excluded. There were 56 respondents who were ineligible due to missing age (*n* = 37), being under the age of 18 years (*n* = 15), or not living, working, or receiving healthcare in Arkansas (*n* = 4). Sixteen respondents did not complete the survey past the eligibility questions. Finally, 68 respondents indicated they had utilized telehealth prior to the onset of the COVID-19 pandemic. As the focus of this particular study is the first use of telehealth during the COVID-19 pandemic, these surveys were removed, leaving a final analytic sample of 1,137 respondents.

### 2.4. Data Analysis

The outcome of interest, telehealth utilization during the COVID-19 pandemic, was determined by the question “*How have you used telehealth?*” Telehealth was defined as “using video to allow you and your doctor to talk without being in the same room.” Respondents were coded as *nonutilizers* if they indicated they had never used telehealth and *utilizers* if they had not utilized telehealth before the pandemic but indicated first using telehealth during the pandemic.

#### 2.4.1. Predisposing Domain

Predisposing factors include sociodemographic characteristics and health beliefs. Age in years was included as a continuous variable. Sex was dichotomized as male/female. Race/ethnicity was categorized as White, Black, Hispanic, and any other racial/ethnic category (due to the low number of responses in the American Indian/Alaska Native, Native Hawaiian/Pacific Islander, and Asian categories). Education was categorized as less than a high school education, completed high school, and more than a high chool education. Marital status was dichotomized as unmarried/nonpartnered or married/partnered.

COVID-19-specific health beliefs were included in the predisposing domain. Perceived susceptibility to COVID-19 scale was created by summing responses from the questions: (1) “*What do you think your chances are of getting COVID-19?*”; (2) “*What do you think your chances are of dying from COVID-19?*”; (3) “*I know how to protect myself from COVID-19.*”; and “*For me, avoiding an infection with COVID-19 in the current situation is…?*” (*α* = 0.52). The perceived susceptibility scale ranged from 0 to 8, with higher scores indicating higher perceived susceptibility to COVID-19. The COVID-19 fear scale was created by summing responses from questions asking the level of concern over various issues related to the COVID-19 pandemic, including concerns over finances, being infected or infecting others, the overall economy, and returning to normalcy postpandemic (*α* = 0.86). The COVID-19 fear scale ranged from 0 to 42, with higher scores indicating a higher level of fear.

#### 2.4.2. Enabling Domain

Enabling factors include socioeconomic factors and access to care. Income was categorized as less than $25,000, $25,000 to $49,999, and $50,000 or more. Several variables were dichotomized: employment (employed for wages/not employed for wages), insurance coverage (yes/no), and if a stimulus check was received (yes/no). Confidence in medical knowledge was dichotomized as confident/unconfident, using the question “*How confident are you filling out medical forms by yourself?*” Usual source of care was categorized as none, one provider, or two or more providers. Finally, internet devices were a continuous variable of the number of internet-capable devices in the respondent's home.

#### 2.4.3. Need for Care Domain

Factors in this domain included preexisting and newly diagnosed conditions and other reasons to seek healthcare. There were five continuous variables: the number of (1) physical health conditions (range 0-15); (2) mental health conditions (range 0-2); (3) COVID-19 stressors, including health concerns and access to medical supplies (range 0-13); (4) COVID-19-induced changes in healthcare, including changes in access or cancellation of surgeries (range 0-4); and (5) COVID-19-related barriers to care, including the need to quarantine/isolate or avoid clinics and hospital waiting rooms (range 0-9). The respondent's perceived change in health since the beginning of the pandemic (better/about or same/worse) was also included.

The descriptive statistics report means (*m*) and standard deviations for continuous variables and the frequency and percentages for categorical variables. *t*-tests and Chi Square tests assessed the relationship between telehealth use and each independent variable. Multivariable logistic regression with full information maximum likelihood (FIML) estimation was used to determine the odds (OR) of utilizing telehealth during the COVID-19 pandemic. FIML was used to account for the missing data, and sensitivity tests, using multiple imputation with chained equations, were performed with similar results [35–37]. Analyses were completed using MPlus, and a *p* value of 0.05 or less was considered statistically significant.

## 3. Results

### 3.1. Demographics and Bivariate Analyses

Table 1 describes the respondents and shows bivariate relationships.

#### 3.1.1. Predisposing Factors

Utilization of telehealth was significantly associated with several factors from this domain in the bivariate analysis: gender (*p* = 0.023), race/ethnicity (*p* = 0.003), educational attainment (*p* = 0.007), confidence filling out medical forms (*p* = 0.024), and the COVID-19 fear scale (*p* = 0.001). More women (37.3%) reported the utilization of telehealth than men (29.7%). Fewer Black Arkansans (22.4%) utilized telehealth compared to other racial/ethnic groups. Telehealth utilizers scored lower (*m* = 15.73) on the COVID-19 fear scale compared to nonutilizers (*m* = 17.52). A higher percentage of respondents (43.9%) who reported being confident in their medical knowledge used telehealth, compared to those who were not confident (30.0%). Utilizers were slightly older (*m* = 48.8) than nonutilizers (*m* = 48.1), but there was no significant difference in age. Marital status and the COVID-19 susceptibility scale were not significant.

#### 3.1.2. Enabling Factors

Insurance status (*p* = 0.003) and having a current healthcare provider (*p* < 0.001) were enabling factors associated with telehealth utilization in the bivariate analysis. A higher percentage of people with insurance (7.2%) reported using telehealth during the COVID-19 pandemic, compared to those without insurance (2.7%). A higher percentage of those with more than one regular healthcare provider (53.1%) reported using telehealth compared to those with one (40.7%) or no regular healthcare provider (28.2%).

#### 3.1.3. Need for Care Factors

All of the need factors were associated with telehealth utilization in the bivariate analysis, including the number of physical health (*p* < 0.001) and mental health conditions (*p* < 0.001), the number of COVID-19-related stressors (*p* < 0.001), the number of changes in healthcare delivery (*p* < 0.001) and barriers to healthcare (*p* < 0.001) during the pandemic, and self-rated health during the pandemic (*p* = 0.002). Those who reported telehealth use had more physical (*m* = 2.45) and mental (*m* = 0.89) health conditions compared to those who did not report telehealth use.

### 3.2. Odds of Utilizing Telehealth during the COVID-19 Pandemic

Table 2 presents the results of the multivariable logistic regression predicting the odds of telehealth utilization.

#### 3.2.1. Predisposing Factors

Race/ethnicity was associated with telehealth utilization; Black Arkansans had lower odds of reporting telehealth utilization during the COVID-19 pandemic compared to White Arkansans (OR = 0.57, CI [0.33, 0.96]). Educational attainment was associated with telehealth utilization, with those having a igh chool diploma or less education having lower odds of telehealth utilization compared to those with greater than a high school education (OR = 0.45, CI [0.25, 0.83]). Age, sex, marital status, confidence in completing medical forms, COVID-19 fear, and COVID-19-perceived susceptibility were not associated with telehealth utilization.

#### 3.2.2. Enabling Factors

The only enabling factor associated with the utilization of telehealth was having more than one current healthcare provider. Individuals who reported more than one current healthcare provider had 2.33 times higher odds of reporting the utilization of telehealth compared to those who did not report having a current healthcare provider (CI = 1.30, 4.18). Income, employment status, health insurance status, the number of internet-capable devices in the home, and the receipt of a stimulus check were not associated with telehealth utilization.

#### 3.2.3. Need for Care Factors

The number of physical and mental health conditions was associated with the utilization of telehealth; for each additional physical health condition an individual reported, the odds of reporting the utilization of telehealth increased by 1.12 (CI = 1.00, 1.25), and for each additional mental health condition reported, the odds of telehealth utilization increased by 1.53 (CI = 1.24, 1.88). For each additional change in healthcare delivery related to COVID-19, the odds of using telehealth increased by 3.49 (CI = 2.78, 4.38). COVID-19-related stress, barriers to care, and changes in health status during COVID-19 were not associated with the utilization of telehealth.

## 4. Discussion

We hypothesized that the three domains of Andersen's Model—predisposing, enabling, and need for care—would influence the utilization of telehealth during the COVID-19 pandemic. Individually, many of the factors that make up each domain did have an association with the utilization of telehealth, partially supporting our hypotheses. However, contrary to our hypotheses, some of the associations were not significant in the final multivariate model examining telehealth utilization during the pandemic.

### 4.1. Predisposing Factors

In the final multivariable model, Black Arkansans and Arkansans with lower educational attainment were less likely to report the utilization of telehealth. These results partially support hypothesis H1a and are consistent with prepandemic research [38]. Prior work has demonstrated a lack of access to, and utilization of, healthcare has left underresourced populations more vulnerable to the effects of COVID-19, leading to serious illnesses, hospitalizations, and deaths. This finding is an indication of ongoing health disparities by race and socioeconomic class, resulting in the disproportional number of deaths from COVID-19 in these communities [39–41]. Research has shown that the use of telehealth has the ability to reduce health disparities and close disparities in care for underresourced groups [10, 42].

There are barriers, however, to the utilization of telehealth in these communities. Lack of internet connection or slow internet connections (e.g., through cellular networks), unfamiliarity with software or compatibility issues with older equipment, and scheduling conflicts with shift work have all been previously cited as barriers to telehealth utilization in underresourced communities [14]. Moreover, a lack of trust in medical technology may play a role in the utilization of telehealth for Black individuals and those with lower educational attainment [14–16]. Prior research has shown that Black Americans and those with lower levels of education have more concerns regarding confidentiality, privacy, and the lack of physical presence of a provider in telehealth visits than other groups [15, 16]. Our work suggests that the restrictions imposed on face-to-face healthcare services by the pandemic were insufficient to facilitate use of telehealth by these two populations. Future research will need to focus on understanding barriers, including issues of trust in the medical system, found in these populations, ideally through community-based partnerships [43, 44].

The results indicate there was no significant association between age, gender, or marital status and telehealth utilization. As more Americans started to use telehealth for the first time due to the COVID-19 pandemic, demographic predisposition to telehealth utilization might have become less pronounced. Age has been a concern for the use of telehealth. Older Americans have previously reported barriers to the utilization of telehealth, including the availability of internet-capable devices, technical literacy and technical support from family and friends, trust in the security and safety of telehealth services, and cost, as telehealth care had been ineligible for reimbursement from Medicare prior to the COVID-19 pandemic [9]. However, the necessity of telehealth during COVID-19 may have been the catalyst for older Americans, aided by the change in Medicare rules to cover telehealth services, to try telehealth services for the first time.

Perceived susceptibility, COVID-19 specific fears, and confidence in medical knowledge were not associated with the utilization of telehealth in the multivariable models, contrary to hypothesis H1b. Although perceived susceptibility and COVID-19 specific fears were conceptualized as health beliefs in the research presented here, it may be that these scales are indicators of health anxiety and more closely related to the perception for the need for healthcare [45]. It may also be that fears of infection and susceptibility of infection are normalized in a global pandemic, allowing for the rationalization of fears and COVID-19 susceptibility as normal/expected and, in turn, not driving the need to avoid healthcare settings and accounting for the null results for hypothesis H1b [45, 46]. Future research should consider the connection between health anxiety and the utilization of telehealth during the COVID-19 pandemic.

### 4.2. Enabling Factors

One barrier to the utilization of telehealth has been cost, with past research showing higher income, being employed, and having health insurance being associated with the utilization of telehealth [9]. Stimulus payments received as part of the government's response to the COVID-19 pandemic have not previously been studied in the utilization of telehealth but are considered a way to augment income. However, contrary to hypothesis H2a, our study did not show the same associations, potentially due to changes in the financing of healthcare during the COVID-19 pandemic. Many screening services for COVID-19 symptoms took place via phone or internet free of charge, which may have encouraged more people to utilize telehealth services who would normally not do so. Online healthcare services, many with a one-time fee, have become more prevalent as technology has improved, allowing for access to both physical and mental healthcare in nontraditional ways. Future studies may want to consider the types of telehealth services people are accessing and the level of cost associated with each.

Having more than one regular provider was the only enabling factor associated with the utilization of telehealth in the final multivariate model, partially supporting hypothesis H2b. It may be that having more than one provider increases access and opportunities to use telehealth services. Prior work has shown that having a regular care provider encourages individuals to seek out care and to avoid delaying care, which may help to explain the increase in utilization of telehealth services in this group [47]. During the COVID-19 pandemic, the use of telehealth may have been one way to avoid delaying needed primary care services.

### 4.3. Need for Care Factors

Many of the need for care factors were associated with the utilization of telehealth, partially supporting hypothesis H3. For Arkansans in our study, those with more physical and mental health conditions were more likely to have utilized telehealth services, which may indicate that those who need the care the most are more willing to try new ways to access it.

Surveys conducted by the Centers for Disease Control and Prevention (CDC) in June 2020 have demonstrated over 40% of respondents have delayed or avoided care during the pandemic, which is concerning over and above the pandemic itself [48]. Prior to the pandemic, rates of telehealth use for routine care were low even among facilities who had robust telehealth programs [32]. However, the reported number of changes in the delivery of healthcare due to COVID-19 may also be driving this change (e.g., closing clinics and canceling elective procedures), as an increase in the number of changes reported by individuals increased the odds of utilization of telehealth during the pandemic. Future research should consider the efforts made by providers during the COVID-19 pandemic in order to understand the efficacy of different approaches to facilitating telehealth utilization.

### 4.4. Limitations

There are a few limitations to keep in mind when interpreting the results of this study. Participants were self-selected into the study, and the sample is predominately White, female, and highly educated; therefore, the sample may not be representative of all of Arkansas and may not be generalizable to larger populations. All responses were self-reported and do carry a risk of social desirability bias. This limitation is reduced through the use of validated instruments, and, although social desirability may play a role, prior work demonstrated limited effects even for sensitive questions (e.g., substance use) [49].

## 5. Conclusions

Using Andersen's Model, our results show that disparities exist in Arkansans' utilization of telehealth services during the COVID-19 pandemic. In particular, being Black or having an education of high school or less was associated with lower odds of telehealth use, whereas having more than one healthcare provider and more chronic physical or mental health conditions was associated with higher odds of telehealth use. Future research should explore the disparities in telehealth utilization and how telehealth might be used to address disparities in access to healthcare in the context of a historic pandemic.

## Figures and Tables

**Table 1:** Characteristics of sample, overall, and telehealth utilization (*n* = 1,137).

	Nonutilizers			Utilizers			*t* test or chi square test p value
	Mean/proportion	SE	95% CI	Mean/proportion	SE	95% CI	
*Predisposing factors*							
Age (in years) (*n* = 1,137)	48.1	0.58	47.0, 49.3	48.8	0.78	47.3, 50.3	0.488
Gender (*n* = 1,135)							**0.023**
Male	196 (70.3)	0.03	64.6, 75.3	83 (29.7)	0.03	24.7, 35.4	
Female	537 (62.7)	0.02	59.4, 65.9	319 (37.3)	0.02	34.1, 40.6	
Race/ethnicity (*n* = 1,132)							**0.003**
White	541 (62.4)	0.02	59.1, 65.6	326 (37.6)	0.02	34.4, 40.9	
Black	121 (77.6)	0.02	70.3, 83.4	35 (22.4)	0.02	16.6, 29.7	
Hispanic	43 (59.7)	0.06	48.1, 70.4	29 (40.3)	0.06	29.6, 51.9	
Other race or ethnicity	24 (64.9)	0.08	48.4, 78.4	13 (35.1)	0.08	21.6, 51.6	
Educational attainment (*n* = 1,134)							0.007
High school or less	108 (78.3)	0.04	70.6, 84.4	30 (21.7)	0.04	15.6, 29.4	
College 1-3 years or tech school	203 (65.3)	0.03	59.8, 70.4	108 (34.7)	0.03	29.6, 40.2	
College degree or more	420 (61.3)	0.02	57.6, 64.9	265 (38.7)	0.02	35.1, 42.4	
Marital status (*n* = 1,135)							0.070
Unmarried/nonpartnered	285 (67.9)	0.02	63.2, 72.2	135 (32.1)	0.02	27.8, 36.8	
Married or partnered	447 (62.5)	0.02	58.9, 66.0	268 (37.5)	0.02	0.34.0, 41.1	
Confidence filling out medical forms (*n* = 941)							**0.024**
Not confident	49 (70.0)	0.06	58.3, 79.6	21 (30.0)	0.06	20.4, 41.7	
Confident	489 (56.1)	0.02	52.8, 59.4	382 (43.9)	0.02	40.6, 47.2	
COVID-19 susceptibility scale (*n* = 771)	4.28	0.04	4.19, 4.36	4.36	0.06	3.25, 4.47	0.211
COVID-19 fear scale (*n* = 933)	17.52	0.36	16.81, 18.23	15.73	0.39	14.96, 16.50	**0.001**
*Enabling factors*							
Annual income (*n* = 907)							0.358
Under $25,000	112 (58.6)	0.04	51.5, 65.4	79 (41.4)	0.04	34.6, 48.5	
Over $50,000	166 (61.5)	0.03	55.5, 67.1	104 (38.5)	0.03	32.9, 44.5	
Over $75,000	250 (56.1)	0.02	51.4, 60.6	196 (43.9)	0.02	39.4, 48.6	
Employment status (*n* = 978)							0.957
Employed for wages	404 (58.6)	0.02	54.9, 62.3	285 (41.4)	0.02	37.7, 45.1	
Not employed for wages	170 (58.8)	0.02	53.0, 64.4	119 (41.2)	0.03	35.6, 47.0	
Health insurance status (*n* = 934)							**0.003**
Insured	492 (92.8)	0.01	90.3, 94.7	38 (7.2)	0.01	5.3, 9.7	
Uninsured	393 (97.3)	0.01	95.1, 98.5	11 (2.7)	0.01	1.5, 4.9	
Count of internet devices in the home (*n* = 872)	2.75	0.03	2.69, 2.81	2.83	0.03	2.77, 2.89	
Current healthcare provider (*n* = 972)							**<0.001**
No	79 (71.8)	0.04	62.7, 79.4	31 (28.2)	0.04	20.6, 37.3	
Yes, only one	319 (59.3)	0.02	55.1, 63.4	219 (40.7)	0.02	36.6, 44.9	
Yes, more than one	135 (46.9)	0.03	41.2, 52.7	153 (53.1)	0.03	47.3, 58.8	
Stimulus check received (*n* = 884)							0.942
No	115 (59.0)	0.02	51.9, 65.7	80 (41.0)	0.02	37.9, 44.8	
Yes	456 (58.7)	0.04	55.2, 62.1	321 (41.3)	0.04	34.3, 48.1	
*Need for care factors*							
Count of chronic physical health conditions (*n* = 944)	1.72	0.07	1.58, 1.87	2.45	0.09	2.26, 2.63	**<0.001**
Count of mental health conditions (*n* = 994)	0.47	0.03	0.41, 0.54	0.89	0.04	0.80, 0.98	**<0.001**
Count of COVID-19-related stressors (*n* = 1,137)	4.58	0.10	4.38, 4.79	5.73	0.13	5.49, 5.99	**<0.001**
Count of changes in healthcare related to COVID-19 (*n* = 1,137)	0.44	0.03	0.39, 0.48	1.41	0.05	1.32, 1.50	**<0.001**
Count of barriers to care related to COVID-19 (*n* = 865)	1.28	0.07	1.14, 1.43	1.93	0.09	1.75, 2.12	**<0.001**
Self-rated health during COVID-19 compared to before (*n* = 959)							**0.002**
Worse	41 (55.4)	0.06	0.44, 0.66	33 (44.6)	0.06	0.34, 0.56	
About the same	434 (61.6)	0.02	0.58, 0.65	270 (38.4)	0.02	0.35, 0.42	
Better	86 (47.5)	0.04	0.40, 0.55	95 (52.5)	0.04	0.45, 0.60	

**Table 2:** Odds of adopting telemedicine during the COVID-19 pandemic (*n* = 1,137).

	Odds ratio	Std. err.	95% conf. interval	*p* value
*Predisposing factors*				
Age (in years)	0.996	0.006	0.98, 1.01	0.505
Gender				
Female	1.13	0.189	0.78, 1.64	0.513
Race/ethnicity				
Black	0.57	0.27	0.33, 0.96	**0.033**
Hispanic	1.45	0.32	0.77, 2.73	0.254
Other race or ethnicity	1.01	0.45	0.42, 2.42	0.074
Educational attainment				
High school or less	0.45	0.31	0.25, 0.83	**0.010**
College 1-3 years or tech school	0.73	0.19	0.50, 1.07	0.102
Marital status				
Married or partnered	1.36	0.17	0.97, 1.91	0.08
Confidence filling out medical forms				
Confident	1.78	0.34	0.92, 3.40	0.09
COVID-19 susceptibility scale	1.06	0.09	0.88, 1.26	0.550
COVID-19 fear scale	0.99	0.01	0.97, 1.02	0.404
*Enabling factors*				
Annual income				
Under $25,000	0.76	0.26	0.46, 1.27	0.300
Over $50,000	0.90	0.20	0.60, 1.34	0.594
Employment status				
Not employed for wages	0.93	0.20	0.63, 1.38	0.708
Health insurance status				
Uninsured	0.45	0.43	0.19, 1.05	0.064
Count of internet devices in the home	1.12	0.13	0.87, 0.56	0.382
Current healthcare provider				
Yes, only one	1.54	0.29	0.88, 2.69	0.131
Yes, more than one	2.33	0.30	1.30, 4.18	**0.005**
Stimulus check received				
Yes	1.09	0.21	0.72, 1.65	0.686
*Need for care factors*				
Count of chronic physical health conditions	1.12	0.06	1.00, 1.25	**0.043**
Count of mental health conditions	1.53	0.11	1.24, 1.88	**<0.001**
Count of COVID-19-related stressors	1.03	0.04	0.96, 1.10	0.391
Count of changes in healthcare related to COVID-19	3.49	0.12	2.78, 4.38	**<0.001**
Count of barriers to care related to COVID-19	1.00	0.06	0.89, 1.12	1.00
Self-rated health during COVID-19 compared to before				
Worse	1.36	0.28	0.79, 2.35	0.274
Better	1.12	0.21	0.75, 1.68	0.581

## Data Availability

The deidentified data underlying the results presented in this study may be made available upon request from the corresponding author, Dr. Pearl A. McElfish, at pamcelfish@uams.edu. The data are not publicly available in accordance with funding requirements and participant privacy.
